# Risk Assessment in Peacekeeping: Are Visual Sparse Models Transparent?

**DOI:** 10.1111/risa.70351

**Published:** 2026-09-03

**Authors:** Niklas Keller, Uwe Czienskowski, Harald Schaub, Konstantinos V. Katsikopoulos

**Affiliations:** ^1^ Harding Centre for Risk Literacy Berlin Germany; ^2^ Centre for Adaptive Rationality Max Planck Institute for Human Development Berlin Germany; ^3^ Department of Information Technologies Max Planck Institute for Human Development Berlin Germany; ^4^ Department of General Psychology and Methodology University of Bamberg Bamberg Germany; ^5^ IABG (Industrieanlagen‐Betriebsgesellschaft mbH) Ottobrunn Germany; ^6^ Centre for Behavioural Experimental Action and Research, Department of Decision Analytics and Risk University of Southampton Business School Southampton UK

**Keywords:** classification, fast‐and‐frugal heuristics, peacekeeping, sparse decision trees, transparency

## Abstract

Decision and risk analysis tools must be accurate and transparent. Classification trees, especially sparse ones, and other visual models, such as scorecards or risk tables, have been claimed to strike this balance. There is, however, little empirical evidence for the transparency of such models. We derive relevant hypotheses and test them in a controlled laboratory experiment with an ecologically valid, high‐risk, critical task: threat classification in peacekeeping. Three classification models are studied: a complete tree, a sparse (specifically, fast‐and‐frugal) tree, and a risk table. To focus on transparency, all three models make identical classifications and thus have equal accuracy. We assess and score three aspects of transparency for each model: time required to learn to a strict criterion, accuracy of application under time pressure, and accuracy in a delayed surprise memory recall test. In a between‐participants design, the fast‐and‐frugal tree is learned more quickly, applied more accurately, and recalled more accurately than the complete tree and the risk table; all statistical effect sizes are large. The recall accuracy of the fast‐and‐frugal tree is, in contrast to the other two models, robust to individual differences in statistical numeracy and risk literacy. In sum, the results of the experiment, together with reflection on limitations and challenges, plus theoretical arguments, suggest that sparse trees might serve as a reasonable benchmark and starting point for designing transparent support for risk assessment.

## 1 Introduction: Visual Sparse Models for Transparent Risk Assessment?

Practitioners require tools that are accurate and transparent. For example, in high‐stakes, risky, emergency situations, such as classifying vehicles approaching a checkpoint as civilian (low risk) or suicide attacker (high risk), models must balance misses and false alarms, in an understandable, speedy, and efficient manner. An idea that has stood the test of time is Breiman et al. (1984, 2001) proposal of employing *visual* models, such as decision trees. Many fields that aim to support high‐risk decision‐making touch on such issues, such as machine learning, operations research, naturalistic decision making, and risk analysis of emergency situations.

Machine learning studies *sparse* models that have a small number of informative variables; a property that can, under some conditions, increase predictive accuracy (Rish and Grabarnik 2014). Bertsimas and Dunn (2017) assumed that trees are transparent and developed optimization methods for inducing sparse trees. Xin et al. (2022) showed how to compute the set of almost‐optimal sparse trees and Wang et al. (2022) constructed a visualization tool for exploring this set which may contain thousands of trees.

Rudin (2019) and Rudin et al. 2022) explicitly argued that sparsity can increase what she calls interpretability because it allows to better understand how the variables combine to generate a prediction, and she proposed optimization methods for deriving sparse trees and sparse linear models (linear models can be represented visually as scorecards or tables). Herzog and Franklin (2024) reinforced the point that trees are inherently interpretable—trees do not require further interpretation as deep neural networks do—and pointed out that such models have been independently developed in the behavioral and decision sciences.

Indeed, in operations research, Todd (2007) pointed out that, on the job, people typically use a few pieces of information and that such models can lead to superior decisions. As an illustration, Keller and Katsikopoulos (2016) used cognitive task analysis (Vicente 1999) to interview military personnel involved in peacekeeping and derived a three‐attribute tree that classified vehicles approaching a military position as hostile or not, reducing false alarms by 60% compared to unaided soldiers.

Katsikopoulos et al. (2020) provided a theory of a type of sparse classification trees, called fast‐and‐frugal trees, that are predictively accurate across domains including healthcare, politics, and finance; and, as Bertsimas and Dunn (2017), Rudin (2019), and Rudin et al. 2022), pronounced fast‐and‐frugal trees transparent in being simple to understand, execute, and teach to others. Gigerenzer (2024) proposed that a host of sparse models motivated by principles of human psychology, which he labeled psychological‐AI models, can achieve accuracy and transparency. In behavioral operations research (Kunc et al. 2016) lingo, the claim is that decision makers and risk analysts would liaise well with visual sparse models and integrate them into workflows.

Naturalistic decision making has since long focused on high‐risk situations but the models developed are rarely simple nor transparent and usually require a significant degree of domain expertise. For example, as Travadel et al. (2017) showed for the Fukushima Dai Ichi nuclear plant disaster, assessment, and management of extreme risks might require tacit expert knowledge and intuition. Practitioners’ rejection of formal models that require too much information is a regular motif of naturalistic decision making (Zsambok and Klein 1997).

But there are exceptions where visual sparse models have been studied in naturalistic settings. As Keller et al. (2010) advocated, there is room for impactful applications of visual sparse models to high‐risk situations. Notable instances include Snook, Taylor and Bennell's (2004) visual circle heuristic for geographic profiling of serial offenders, and Cook's (2011) fast‐and‐frugal tree used by the New York City Fire Department to triage victims and allocate healthcare at the 9/11 site.

The hunt for models that are usable by practitioners in machine learning, operations research and naturalistic decision making, converges with the risk analysis of emergency situations. A theme of this work is the development of behaviorally informed interventions, so that these interventions are more transparent to practitioners. Examples include techniques and frameworks for countering terrorism and biosecurity threats (Bhashyam and Montibeller 2016; Montibeller et al. 2020). Some, though not all, of this strand of risk analysis research develops models. In contrast to some of the research discussed above, however, risk analysis models are not always designed to be simple or, more specifically, sparse (Zawadzki et al. 2022).

Overall, there are strong reasons, across disciplines, for studying visual sparse models. Might such models indeed strike the required balance between accuracy and transparency? Unfortunately, as Pande et al. (2021) point out, the empirical evidence for the claim that sparse models are transparent is, well, sparse (pun intended). There is, however, some empirical evidence for related claims. There is consensus (Vessey 1991; Coll et al. 1994; Huysmans et al. 2011; though see Subramanian et al. 1992) that people can apply visual models, such as trees and tables, fast and accurately. Poursabzi‐Sangdeh et al. (2021) found that tabular representations of sparse linear models can also be applied accurately.

In sum, one could piece together a case for endorsing visual sparse models for decision and risk analysis support. On the other hand, the supporting evidence is not yet strong enough, specifically for high‐risk, critical domains. Our study is designed to help close this gap.

There are at least four important aspects for which there is a lack of empirical basis: First, the tasks studied previously are typically individual consumer decisions, such as buying DVDs and apartments or deciding whether to approve loans. Such decisions are important, but they might work quite differently from, say, emergency healthcare or peacekeeping, in which the stakes are much higher. Second, the dependent variables typically studied refer only to some aspects of transparency, such as correctly applying the tools. But transparency includes other aspects too, such as mastering tools quickly and reproducing them accurately. Third, previous studies have not tackled tasks that are difficult to perform on the job under time pressure, psychological stress, and noisy information. And lastly, different levels of sparsity have been investigated for linear models, but not for decision trees.

The present study addresses these issues. We run a controlled laboratory experiment within the context of a high‐risk, time‐pressured, critical task in peacekeeping, namely, classifying oncoming vehicles as civilians or potential threats. This choice of task is a strategic one. Firstly, peacekeeping is a very high‐stakes operation. Second, vehicle classification is a high‐risk, critical task within peacekeeping, often having to be executed under time pressure and psychological stress. Third, the task is difficult: an analysis of over 1000 reports from Afghanistan checkpoints (Keller and Katsikopoulos 2016) revealed many false alarms (204 civilians injured or killed) and no vehicle‐borne suicide attacks successfully averted. Finally, a visual sparse model (a fast‐and‐frugal tree) has already been developed and used by armed forces for their pre‐deployment training (Keller et al. 2013).

The rest of the article is organized as follows. Section 2 defines the concepts of the study—fast‐and‐frugal and complete trees, sparsity, and transparency; it also includes a review of the literature on the transparency of visual sparse models such as trees and tables, aimed at developing precise, empirically testable hypotheses. The subsequent section provides the details of the experiment, such as participants, materials and procedure, and design. Section 4 provides the empirical results, with emphasis on statistical analyses. Section 5 concludes by synthesizing this empirical study with previous studies and theoretical arguments to make suggestions for the design of transparent support for risk assessment.

## 2 Concepts, Literature, and Hypotheses

### 2.1 Concepts

**FIGURE 1:**
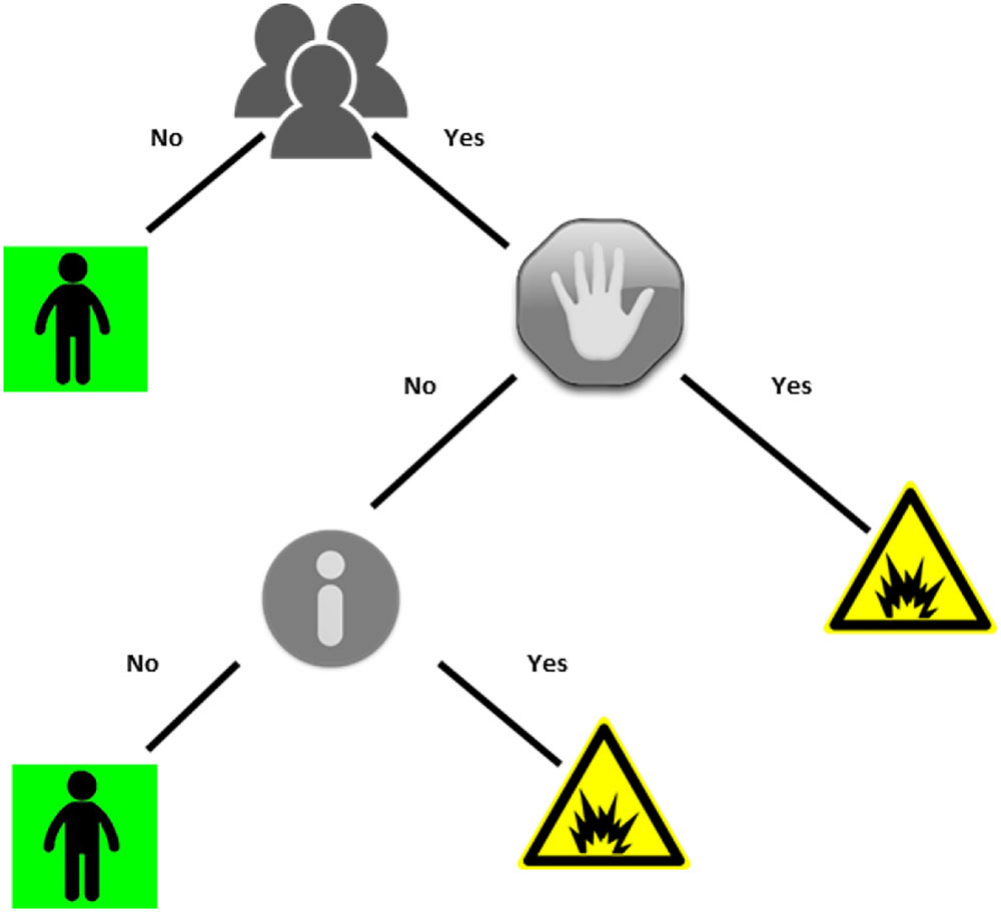
A fast‐and‐frugal tree for classifying vehicles approaching checkpoints (adapted from Keller and Katsikopoulos 2016). A small number of attributes—here three—are queried in a fixed order. After each query there is at least one exit, so it is possible to make a classification without considering all attributes, and even considering only one.

**FIGURE 2:**
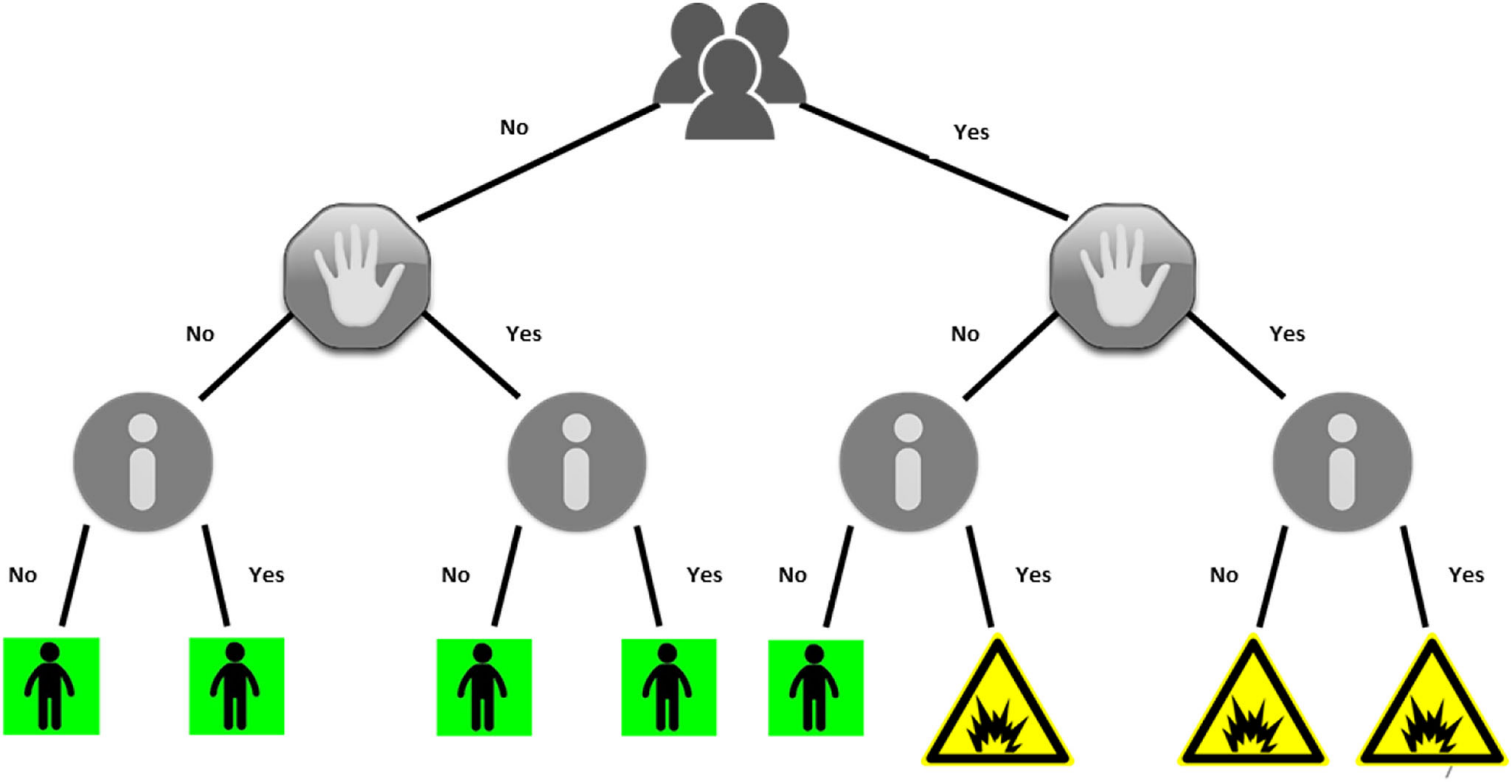
A complete tree that uses the same attributes in the same order and makes the same classifications as the Figure 1 fast‐and‐frugal tree. Unlike the fast‐and‐frugal tree, however, the full tree always uses all attributes before making a classification.

In the remainder of the article, we consider one type of decision: classifications. Classification is a key activity in important fields such as healthcare as when deciding whether a patient is at a high risk of having ischemic heart disease (Green and Mehr 1997), finance as when deciding whether to approve a client for a loan (Lessmann et al. 2015), and so on. Here, we focus on classification tasks with two classes and binary attributes. In our setting, the task is the classification of an oncoming vehicle as either hostile or nonhostile. This classification is based on attributes such as number of occupants in the vehicle and whether it complies with the soldiers’ instructions, namely stopping or slowing down, as it approaches the checkpoint. Figure 1 shows the *fast‐and‐frugal* tree proposed by Keller and Katsikopoulos (2016) for this task. Figure 2 shows a more complex classification tree for the same task, called a *complete* tree. Both trees are tested in the experiment. We next define these types of trees and use them to explain the concepts of sparsity and transparency in the context of the study.

The trees in Figures 1 and 2 use the same three attributes. They first ask whether there are multiple occupants in the vehicle, denoted by the people icon at the top level of the trees. They then ask whether the vehicle is slowing down or stopping, denoted by the stop sign icon at the second level. Finally, they ask whether there are other threat cues signifying that the vehicle is a threat (e.g., intelligence information). This is denoted by the exclamation mark icon at the third level. Furthermore, both trees make the same classifications as nonhostile (green person icon) or hostile (yellow danger triangle icon) for each of the eight combinations of the three binary attribute values. For example, both trees classify as nonhostile a vehicle with no other occupant than the driver that stops or slows down and has no other threat cues. The two differ in that fast‐and‐frugal trees allow for a classification each time they consult an attribute, whereas the complete trees make classifications only after consulting all attributes.

A fast‐and‐frugal tree always has an exit after it queries an attribute, and this property is characteristic of fast‐and‐frugal trees and has served as one of their initial defining features (Martignon et al. 2008). For practical purposes, it often makes sense to augment this definition with extra conditions (Katsikopoulos et al. 2020, chapter 2), as in the following:

Definition 1 *A fast‐and‐frugal tree is a classification tree such that it: (i) always has an exit after it queries an attribute (two exits after it queries the last attribute), (ii) has only a “few” attributes (a common default is three attributes), (iii) queries each attribute once, and (iv) does not query multiple attributes together*.

Definition 1 says that a fast‐and‐frugal tree with *n* attributes has *n* levels, where each level has one query on one attribute, and it has *n* + 1 exits. For confirmation see Figure 1.

The formalism of fast‐and‐frugal trees has been inspired by classification rules often used by practitioners in situations of high stakes with limited resources of information, time, and computation. For example, the “simple triage and rapid treatment” tree developed in the eighties and employed by the New York City Fire Department on the morning of the September 11 attacks (Cook 2011), is a fast‐and‐frugal tree, without being named such. The tree is designed to prioritize victims in mass‐casualty incidents, and its first query is whether a person can walk; if yes, this person is immediately classified as not needing urgent help and if no, further queries are made. We argue that fast‐and‐frugal trees are by design sparser and more transparent than complete trees. To explain what this means, we define when one model is sparser and more transparent than another.

Definition 2 *Model A is sparser than model B if (i) A uses a subset of the attributes B uses and (ii) A makes fewer queries for each attribute common with B*.

The condition in part (i) of Definition 2, that a model is sparser when it employs fewer attributes, is taken from Rish and Grabarnik (2014). In linear regression, this might mean that a regularization penalty is used to force some attribute weights to equal approximately zero, and thus to essentially use fewer attributes (Zou and Hastie 2005). The condition in part (ii) of Definition 2, to query each attribute fewer times, is an additional way of making a model sparser. Statistical induction of trees includes pruning modules that satisfy the two conditions (Breiman et al. 1984; Bertsimas and Dunn 2017). Fast‐and‐frugal trees achieve sparsity by querying fewer attributes and each attribute at most once (Definition 1 and Figure 1).

On a particular instance, some attributes might not be queried in a fast‐and‐frugal tree; for example, if the vehicle has two occupants, no further attributes are queried in Figure 1. The complete tree in Figure 2, however, always queries all three attributes before making a classification. Thus, the fast‐and‐frugal tree is sparser that the complete tree.

What is transparency? We employ the following definition (Katsikopoulos et al. 2020, 26):

Definition 3 *Model A is more transparent than model B to a group of users if these users can “better” understand, memorize, execute, and teach A than B*.

Definition 3 integrates ideas from multiple disciplines. In machine learning, Lipton (2018) identifies model transparency with understanding the model and then, similar to Rudin (2019), suggests that to understand a model means to contemplate the model at once. Thus, one should be able to accurately memorize a transparent model, or at least its most important aspects. In cognitive psychology, understanding something is closely related to being able to apply it correctly and explain it effectively to others (Bruner 1979). In operations research and decision analysis, supporting the understanding and execution of models by decision makers is of central concern (Franco and Montibeller 2010).

Other definitions of transparency have been proposed. However, Lipton (2018) cautions against the proliferation and conflation of terms such as explainability, interpretability, trustworthiness, and simulability. Some of these terms often take meanings that are too technical to be useful to decision makers without training in areas such as statistics and computer science. Focusing on operational aspects, our Definition 3 focuses on usability and applicability on the job.

How to operationalize “better” in Definition 3 and compare the transparency of two models? A proxy to model transparency is the model's *size* (Huysmans et al. 2011; Lipton 2018; Katsikopoulos et al. 2018). Intuitive measures of model size incorporate the number of parameters in the model. The number of parameters is related to the number of attributes but the two are not the same. While an attribute might map onto a parameter in a model, the way in which attributes are combined, and other aspects of the model's functional form are also relevant for a model's size. Nevertheless, when comparing the transparency of models of the same class, as two trees, one can focus on the number of parameters, and this is what we will do to develop hypotheses and derive predictions.

**FIGURE 3:**
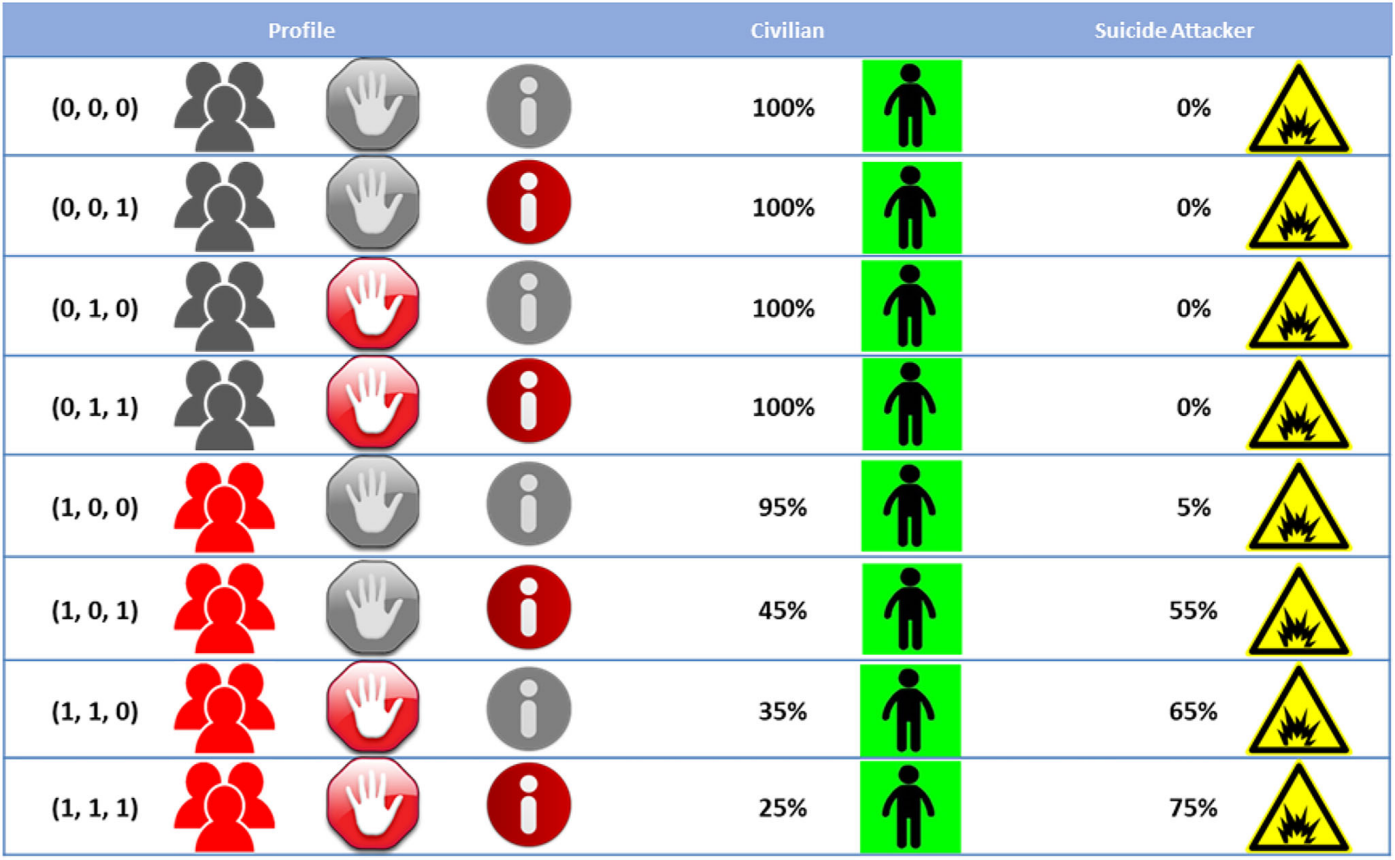
A risk table making the same classifications as the trees of Figures 1 and 2. The table is accompanied by the rule “If the risk is higher than 50%, shoot; otherwise, do not”.

The third model tested in our experiment is the *risk table* presented in Figure 3. This table makes the same classifications with the two trees in Figures 1 and 2 for all eight combinations of attribute values. Similar to the complete tree, the risk table lists explicitly these eight cases. We acknowledge that these design choices—equating the accuracy of all three models and making the classifications of two models explicit—favor experimental control over external validity. Note, however, that risk tables as the one in Figure 3 are indeed used by western militaries and in NATO doctrines to convey threat information and provide decision support to troops (Keller 2013).

### 2.2 Literature

People have been employing trees to support reasoning and decision making since antiquity (Hacking 2007). Another popular type of visual models are tables, in use for about 4500 years (Campbell‐Kelly et al. 2003). The empirical evaluation of the usefulness of trees and tables for processing numerical information has been an active research area since the early twentieth century (Carter 1947).

A central issue in the evaluation of trees and tables is to specify the conditions under which they lead to good human performance, such as fast and accurate reasoning and decision making. A proposal that has stood the test of time is Vessey's (1991) cognitive fit theory, which is rooted in the information processing approach of Simon and his coworkers (Simon 1956; Newell and Simon 1972). According to cognitive fit theory, human performance is fast and accurate if there is a fit between the format in which information is represented and the task at hand.

More specifically, because trees represent information spatially, cognitive fit theory predicts that trees support performance in spatial tasks. Spatial tasks are tasks where associations must be made or relations in the data must be discovered. In the peacekeeping task, a spatial question is whether the tree of Figure 1 would classify as hostile all vehicles that do not stop or slow down (the answer is not all of these vehicles). Cognitive fit theory predicts that users of a tree answer such questions fast and accurately, and this has been borne out, as reviewed by Vessey (1991).

Conversely, because tables represent information symbolically, cognitive fit theory predicts that they support performance in symbolic tasks. Symbolic tasks are tasks where discrete values from the data must be extracted. For example, in the peacekeeping task, a symbolic question is whether the Figure 1 tree would classify as hostile a vehicle with a single occupant not stopping or slowing down and for which no further threat cues are available (the answer is yes). Cognitive fit theory predicts that users of a table answer such questions fast and accurately, and this is so (Vessey 1991).

Nevertheless, cognitive fit theory and the evidence for it have limits (Huysmans et al. 2011). First, the evidence comes from relatively simple studies often asking participants to just locate and read information from a tree or a table. Second, the theory is silent with respect to sparsity and transparency. Third, there is no mention of the importance of individual differences.

Huysmans et al. (2011) revised cognitive fit theory so as to include roles for sparsity, transparency, and individual differences, and provided evidence for this revised theory. Huysmans et al. (2011) proposed that individual differences among model users moderate the effect of the information format‐task fit on human performance, and that the model's size—which relates to sparsity and transparency—affects human performance directly, that is, independently of the format‐task fit. Here we follow this kind of thinking and develop precise, empirically testable hypotheses for the performance of users of the two trees and the table.

### 2.3 Hypotheses

We develop three hypotheses by combining the definition of transparency (Definition 3) with a calculation of the relative transparency of the two trees and the table. It suffices for the calculation to capture the number of parameters in each model. This is so because the two trees belong to the same model class, and the risk table is very similar to the complete tree in that they both explicitly provide classifications for all combinations of attribute values.

The three models are presented here with fixed parameters. The fast‐and‐frugal tree of Figure 1 has four fixed parameters: the attribute queried first (occupants), the exit following this first query (one occupant means nonhostile), the attribute queried second (compliance) and the exit that follows this second query (non‐compliance means hostile). The complete tree of Figure 2 has eight fixed parameters: the class labels (hostile or nonhostile) assigned to each of the eight combinations of attribute values, as does the risk table of Figure 3.

Thus, we hypothesize that the fast‐and‐frugal tree is more transparent than the complete tree and the risk table. Based on Definition 3, one can then put forth the following hypotheses:

Hypothesis 1 The fast‐and‐frugal tree is learned faster than the complete tree and the risk table.

Hypothesis 2 The fast‐and‐frugal tree is applied more accurately than the complete tree and the risk table.

Hypothesis 3 The fast‐and‐frugal tree is recalled more accurately than the complete tree and the risk table.

Furthermore, we measured individual differences along a variable central to understanding risk and probabilistic outcomes: risk literacy. Risk literacy refers to one's practical ability to evaluate and understand probabilistic risks for making skilled and informed decisions. A test measuring this ability, widely and cross‐culturally validated, is the Berlin Numeracy Test for educated samples (Cokely et al. 2012), and we used it.

## 3 Experiment

### 3.1 Participants

We used G‐Power for conducting a power analysis. For a one‐way (model), three‐level (fast‐and‐frugal tree, complete tree, and risk table) ANOVA with a significance level *α* = 0.05 and power threshold *β* = 0.8, the power analysis calculated a minimum of 53 participants per condition, for a total of 159 participants. An additional 9 participants per condition were recruited as buffer by the laboratory staff, bringing the total number per condition to 62. Based on above power analysis, a total of 186 participants were recruited from the participant pool of the Max Planck Institute of Human Development experimental laboratory. As there was no published data quantifying expected effect sizes, a medium effect size of 0.25 was taken to be the minimum practical effect size.

The participants’ mean age was 25.9 years (SD = 4.5, range: 18–38, median = 25). Note that 61% of participants were female. We collected data on military experience and knowledge. Military experience was measured using months active in military service and military knowledge was tested by asking questions on 10 military acronyms relating to escalation‐of‐force incidents; the task was to correctly spell out the acronyms. Just two participants had professional military experience (23 and 72 months), six participants had completed the draft service of nine months, and the remaining 178 participants had had 0 months of experience. Knowledge of military acronyms had a mean score of 0.4 out of 10 (SD = 1.2) and 148 participants identified zero acronyms. As in previous studies using the Berlin Numeracy Test, participant scores, which can range from 0 to 4, were normally distributed, with a mean of 1.9 (SD = 1.1) and median of 2.

### 3.2 Materials and Procedure

The experiment was programmed using the C++ and C# language and the .NET Framework 4.0. The video clips serving as stimuli (see Design sub‐section) were taken from a training scenario for military personnel in Virtual Battle Space 2, a military simulation software package used by the US, UK, German, and 15 other militaries as a virtual training platform. The experiment was double‐blind, being administered by laboratory staff of the Max Planck Institute of Human Development, none of whom were involved in the design of the study. Once staff assigned a participant number, the program automatically assigned a classification model to this participant and the experiment started.

### 3.3 Design

**FIGURE 4:**
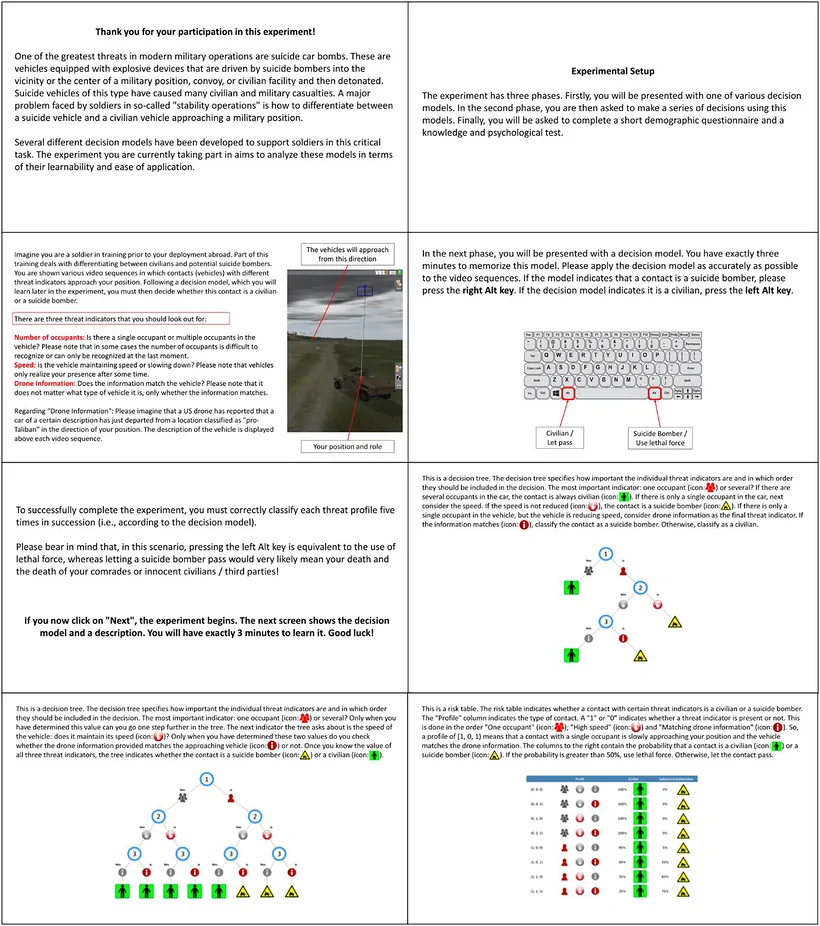
Screens showing the introductory and learning phases of the experiment.

In the experiment, participants first read through and interacted with a series of screens and videos, as shown in Figure 4 (translated into English by the authors). Introductory screens (top row) explaining the purpose and phases of the experiment were followed by screens explaining the setting, layout, role, and task of the participant, as well as the three attributes, the learning criterion to be reached, the operating controls of the experiment, and indicating that participants would next be shown the model for three minutes to learn it (second row and third row, left side). After pressing “continue,” participants were shown the model (third row, right and fourth row), and in three minutes participants continued onto the test phase.

**FIGURE 5:**
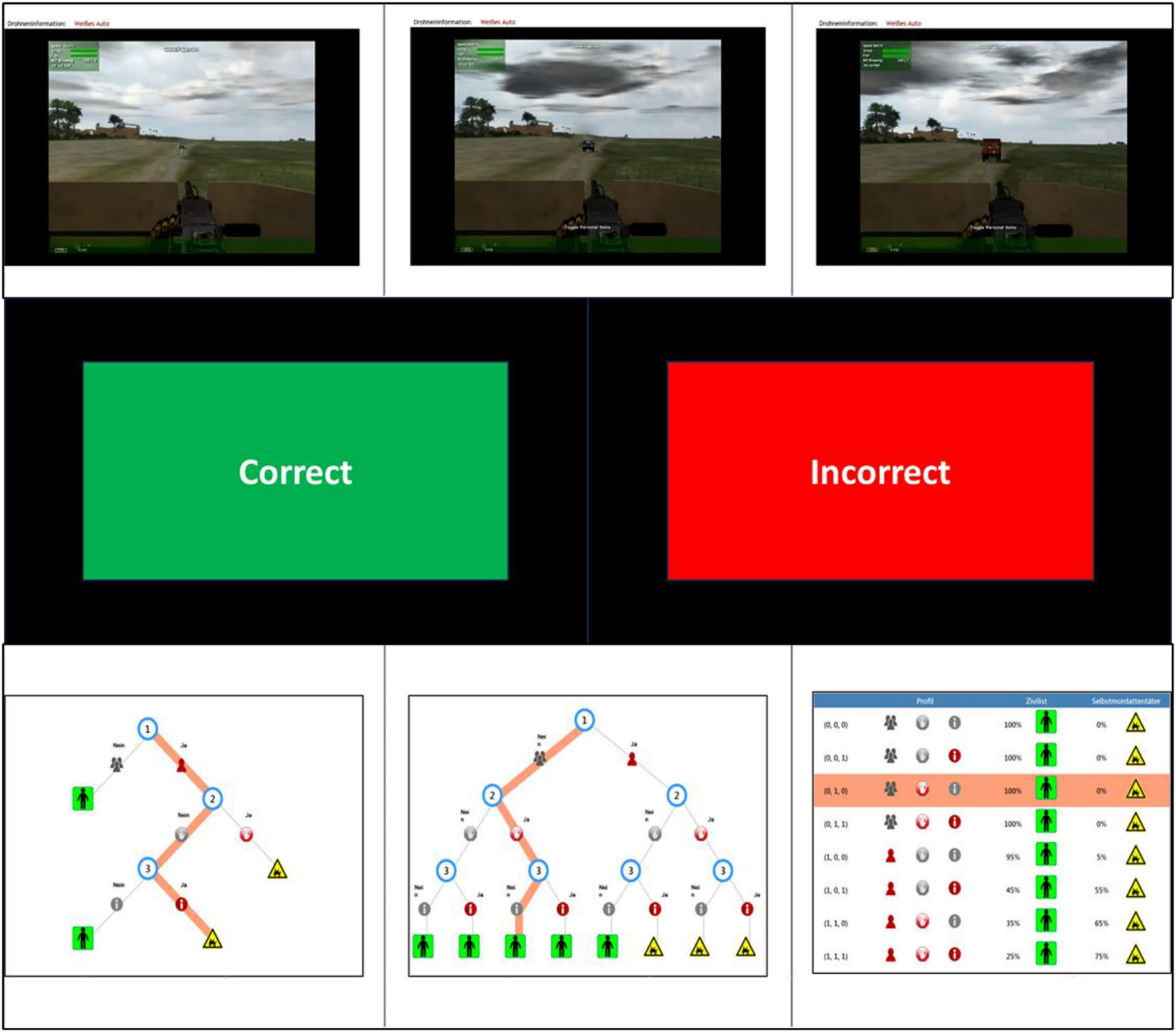
Screens showing the test phase of the experiment (top row), and outcome plus process feedback provided after each classification (respectively, lower two rows).

In the test phase (Figure 5), participants were placed in the role of a soldier guarding a checkpoint and confronted with approaching vehicles (top row) which had to be classified in real time as hostile or nonhostile in accordance with the assigned model. The setup was couched in realistic time pressure: to arrive at the checkpoint, vehicles would take about 27.5 s if they slowed down and 16.7 s if they did not slow down. The cues revealed themselves in a naturalistic way, with the number of occupants usually being only detectable when the vehicle was already relatively close or the vehicle only reacting to the military presence after some time. After the decision had been made by pressing the corresponding button (see Figure 4, second row: “civilian / let pass” or “suicide bomber / use lethal force”), feedback was provided by first providing participants with the outcome of the decision (Figure 5, second row), shown for three seconds, followed by feedback on the correct application of the decision aid by the participant (Figure 5, third row), shown for 15 s. Participants were notified that they could skip this second feedback by clicking the “continue” button and continuing to the next video. The duration for which participants chose to view the second feedback screen was a dependent variable used to measure learning speed. To initiate the next video, a “play” button had to be pressed.

To reach the learning criterion, participants had to apply the assigned classification model correctly five times in a row for all eight combinations of attribute values. If one of the eight attribute combinations was correctly classified five times, the set of stimuli for this combination was removed from the stimulus pool until no more stimuli were left. If a stimulus was incorrectly classified, the counter for this set was reset to zero (this strategy optimizes learning speed according to mathematical models of learning; Katsikopoulos 2000). On each trial, one of the admissible stimuli of the remaining combinations was selected randomly for presentation. The probability of the same stimulus being played repeatedly was reduced by having multiple videos for each combination of attribute values (six videos for the four combinations without “intelligence information” and three videos for the four combinations with “intelligence information”).

In the post‐test phase, which occurred once participants had reached the learning criterion, the following were administered: a demographic questionnaire, the test of military knowledge, the Berlin Numeracy Test, a surprise memory test asking participants to reproduce the model from memory, and finally an open question on whether they applied the model as given or adapted it. The memory test score equaled the proportion of combinations of attribute values that the reproduced model classified as the assigned model. Because the test was administered unannounced about 12–15 min after the learning phase of the experiment and after a cognitive task (the Berlin Numeracy Test), it can be seen to measure delayed recall accuracy.

### 3.4 Predictions

In sum, the (between‐participants) independent variable was the *classification model* assigned to a participant (fast‐and‐frugal tree, complete tree, risk table), and the dependent variables were the (1) *number of experimental trials* required to reach the learning criterion, (2) *feedback viewing time* for each trial, (3) *error rate* in applying the model (across trials), and (4) *memory test score* for the model (done after all trials).

Based on this experimental design, Hypotheses 1–3 were operationalized as the following predictions:

Prediction 1 (a). *Model has an effect on the number of trials: Users of the fast‐and‐frugal tree reach the learning criterion after fewer trials than users of the complete tree and the risk* *Table* *1*. *Model has an effect on feedback viewing time: Users of the fast‐and‐frugal tree have lower times than users of the complete tree and the risk table*.

Prediction 2 *Model has an effect on error rate: Users of the fast‐and‐frugal tree have lower rates than users of the complete tree and the risk table*.

Prediction 3 *Model has an effect on memory test score: Users of the fast‐and‐frugal tree have higher scores than users of the complete tree and the risk table*.

## 4 Empirical Results and Statistical Analyses

Even though we ran the power analysis based on the assumption of conducting parametric ANOVAs, a reviewer rightly pointed out that non‐parametric analyses, such as Kruskal–Wallis and Mann–Whitney *U* tests, as well as survival analyses and mixed‐effects linear models would be more appropriate, given that our data violates distributional assumptions. Under departures from normality, these rank‐based tests are expected to be as powerful as, or more powerful than, the ANOVA on which the power analysis was based (Hodges and Lehmann 1956). Likewise, as the linear mixed models retain all observations and model the within‐cluster covariance structure, they generally yield power equal to or greater than a comparable ANOVA. We therefore regard still the ANOVA‐based a priori power analysis as a reasonable and conservative estimate of the study's sensitivity and consider the planned sample size adequate for the non‐parametric and mixed‐model analyses reported here. We report the results of these new analyses (results were robust to the change). Mixed‐effects models were estimated in R (Version 4.5.1) using the packages lme4 (Version 1.1.37) and lmerTest (Version 3.1.3). Survival analyses were conducted using the survival (Version 3.8.3) and survminer (Version 0.5.1) packages. Figures were produced using ggplot2 (Version 4.0.1). Statistical analyses and result interpretations were checked with Claude Sonnet 4.6 (Anthropic 2025). All outputs were verified and approved by the authors. All data and code are openly available for independent verification and analyses on GitHub and archived on Zenodo at https://doi.org/10.5281/zenodo.20414374, reference number 10.5281/zenodo.20414374.

### 4.1 Outlier Removal

Some participants were removed prior to data analysis. Details are below for each dependent variable.

*Feedback viewing time*: Total of 36 participants apparently did not realize they could proceed to the next trial by pressing “continue” and never reduced their feedback viewing time across the experiment. Those participants were removed from the feedback‐viewing‐time analysis, leaving 49, 54, and 50 fast‐and‐frugal‐tree, complete‐tree and risk‐table users respectively. On the remaining dataset, we scanned for participants further than 1.5 × interquartile range (IQR) from the median (Andre 2022), though this led to no more removals.

*Error rate*: We removed 12 participants who were further than 1.5 × IQR from the median on their average error rate per trial or their total number of errors across trials. This left 58 participants per model.

*Memory test score*: There were no participants further than 1.5 × IQR from the median, leaving 62 participants per model.

Prediction 1a *Fast‐and‐frugal‐tree users reach the learning criterion after fewer trials*.

**FIGURE 6:**
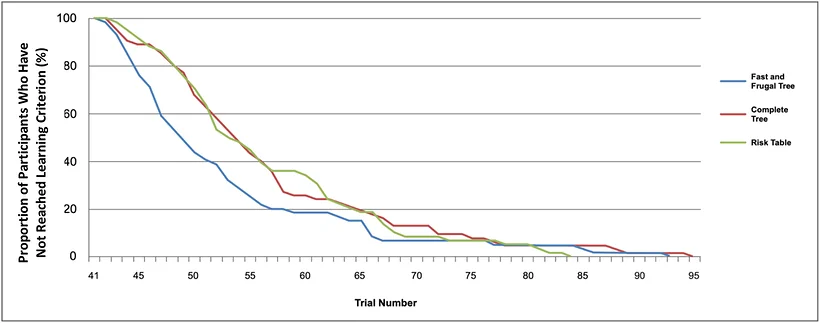
Participants not having reached the learning criterion, as a proportion of users of each model, for all trials after 40.

As participants had to apply the assigned model correctly five times in a row for all eight combinations of attribute values, the learning criterion could only be reached after a minimum of 40 trials. Figure 6 shows, for each model and all trials after 40, the proportion of participants not having reached the learning criterion. Fast‐and‐frugal‐tree users overall reached the learning criterion faster than users of the other models. About 15%–20% more fast‐and‐frugal‐tree users finished the learning phase at any point between trials 45 and 55. Users of the complete tree and the risk table started catching up between trials 55 and 65.

Users of the fast‐and‐frugal tree reached the learning criterion after a median of 47.0 trials, compared to 52.5 and 50.5 trials for the complete tree and risk table users respectively. Table 1 shows this and further descriptive statistics that agree with Figure 1.

**TABLE 1:** Number of trials needed to reach the learning criterion, across participants.

	*N*	Mean	Median	SD	Min	Max
Fast‐and‐frugal Tree	49	52.9	47.0	17.3	40	134
Complete tree	54	55.4	52.5	11.1	41	93
Risk table	50	57.4	50.5	19.1	40	140

Kaplan–Meier estimates of survival times converge with the Table 1 findings, equaling 52.9, 55.4, and 57.4 trials for the fast‐and‐frugal tree, complete tree, and risk table respectively. On the other hand, the corresponding confidence intervals for the medians overlap, [45, 51], [50, 56], and [48, 59], indicating overall moderate differences that might emerge clearly only in particular time windows. This makes sense because eventually all users of all models reached the learning criterion. Hence, we conducted two separate survival analyses, for trials 40–60, 40–55, and Mann–Whitney *U* Tests for all trials after 40.

As Table 2 shows, for trials 40–60 complete‐tree users reached the learning criterion significantly slower than fast‐and‐frugal‐tree users (*p* = 0.028 and hazard ratio = 0.610). On the other hand, the difference in learning speed between fast‐and‐frugal tree and risk table is marginally significant (*p* = 0.060). The overall likelihood‐ratio test narrowly misses conventional significance with *p* = 0.067, and the Nagelkerke *R* ^2^ is 0.037, which denotes a small effect.

**TABLE 2:** Survival analysis (Cox regression) for number of trials needed to reach the learning criterion; trials 40–60.

	*b*	SE	*z*	*p*	HR [95% CI]
Complete tree vs. fast‐frugal tree	−0.494	0.224	−2.20	0.028	0.610 [0.393, 0.947]
Risk table vs. fast‐and‐frugal Tree	−0.430	0.229	−1.88	0.060	0.651 [0.415, 1.019]

Table 3 demonstrates significant learning speed differences for trials 40–55 (consistent with Figure 1), with both *p* values falling below 0.02. The overall likelihood‐ratio test is significant (*p* = 0.011), and the Nagelkerke *R* ^2^ is 0.063, approaching a medium effect.

**TABLE 3:** Survival analysis (Cox regression) for number of trials needed to reach the learning criterion; trials 40–55.

	*b*	SE	*z*	*p*	HR [95% CI]
Complete tree vs. fast‐and‐frugal tree	−0.650	0.234	−2.78	0.006	0.522 [0.330, 0.826]
Risk table vs. fast‐and‐frugal tree	−0.597	0.242	−2.47	0.014	0.550 [0.342, 0.884]

Table 4 shows post hoc Mann–Whitney *U* tests for all trials after trial 40. Consistent with Tables 2 and 3, fast‐and‐frugal‐tree users required significantly fewer trials than complete‐tree and risk‐table users. These overall effects are small or small‐to‐medium. Interestingly, there is no difference between complete tree and risk table (see also Figure 1).

**TABLE 4:** Post hoc Mann–Whitney *U* tests for number of trials needed to reach the learning criterion; after trial 40.

	*W*	*p*	*r*	Effect size
Fast‐and‐frugal tree vs. complete tree	899.0	0.005	0.276	Small‐medium
Fast‐and‐frugal tree vs. risk table	931.0	0.040	0.207	small
Complete tree vs. risk table	1420.5	0.649	0.045	no effect

Finally, note that there were no significant interactions between the results presented above and score on the Berlin Numeracy Test.

Prediction 1b *Fast‐and‐frugal‐tree users have lower feedback viewing times*.

As Table 5 shows, fast‐and‐frugal‐tree users had a median of 136.0 s of cumulative feedback viewing time, compared to 198.3 and 226.6 s for complete‐tree and risk‐table users, respectively. Across participants, we found savings of over 70 and 86 min in total feedback viewing time for the fast‐and‐frugal tree compared to complete‐tree and risk‐table users.

**TABLE 5:** Cumulative feedback viewing times across participants.

	*N*	Mean (s)	Median (s)	SD (s)	Min (s)	Max (s)	Total (min)
Fast‐and‐frugal Tree	49	159.7	136.0	79.3	61.7	414.2	130.4
Complete tree	54	225.1	198.3	116.3	48.0	636.3	202.6
Risk table	50	260.4	226.6	131.9	87.9	612.9	217.0

**FIGURE 7:**
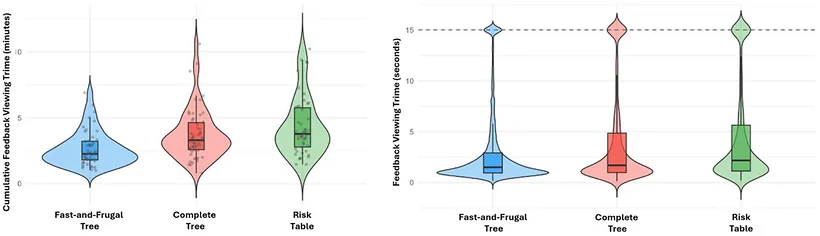
Cumulative (left) and mean (right) feedback viewing times per condition across participants. Dashed line on right plot indicates maximum viewing time (15 s).

Similarly, Figure 7 shows the cumulative feedback viewing time across trials as well as feedback viewing time per trial. Feedback viewing time was lower for the fast‐and‐frugal tree condition (3.62s, SD = 2.10s) than for the complete tree (4.82s, SD = 2.47s) and the risk table conditions (5.31s, SD = 2.76s).

Because feedback viewing times might exhibit variation due to participant and stimulus, we analyzed this dependent variable by a linear mixed‐effects model with crossed random effects for participants and stimuli (8456 observations, 153 participants, 8 stimuli). The results of this analysis are provided in Table 6, showing that complete‐tree and risk‐table users viewed feedback significantly longer than fast‐and‐frugal‐tree users (highly significantly longer for the risk table).

**TABLE 6:** Linear mixed‐effects analysis for feedback viewing time.

	*b*	SE	*t*	df	*p*	exp(*b*)
Fast‐and‐frugal tree (intercept)	7.548	0.063	119.24	150.1	< 0.001	1.896 s
Complete tree	+0.190	0.086	2.22	150.6	0.028	+1.210
Risk table	+0.379	0.087	4.33	150.5	< 0.001	+1.461

Nearly 18% of the total variance is attributable to participants, but only 0.1% is attributable to stimuli. And a likelihood‐ratio test found the overall effect of classification model to be highly significant [*χ* ^2^(2) = 18.05, *p* < 0.001]. A post hoc Kruskal–Wallis test also revealed a highly significant effect of classification model on cumulative feedback viewing time [*H*(2) = 22.137, *p* < 0.001, *η* ^2^ = 0.134].

Finally, Table 7 shows post‐hoc pairwise comparisons using Mann–Whitney *U* tests, confirming that the fast‐and‐frugal tree was learned faster than the complete tree (medium effect) and the risk table (medium‐large effect). Again, there was no significant difference between complete tree and risk table.

**TABLE 7:** Post hoc Mann–Whitney *U* tests for feedback viewing time.

	*W*	*p*	*r*	Effect size
Fast‐and‐frugal tree vs. complete tree	811.0	0.0007	0.333	Medium
Fast‐and‐frugal tree vs. risk table	589.0	< 0.001	0.447	Medium‐large
Complete tree vs. risk table	1116.0	0.129	0.149	No effect

As with the number of trials to reach the learning criterion, there are no significant interactions between the results for feedback viewing time and Berlin Numeracy Test score.

Prediction 2 *Fast‐and‐frugal‐tree users have lower error rates*.

**FIGURE 8:**
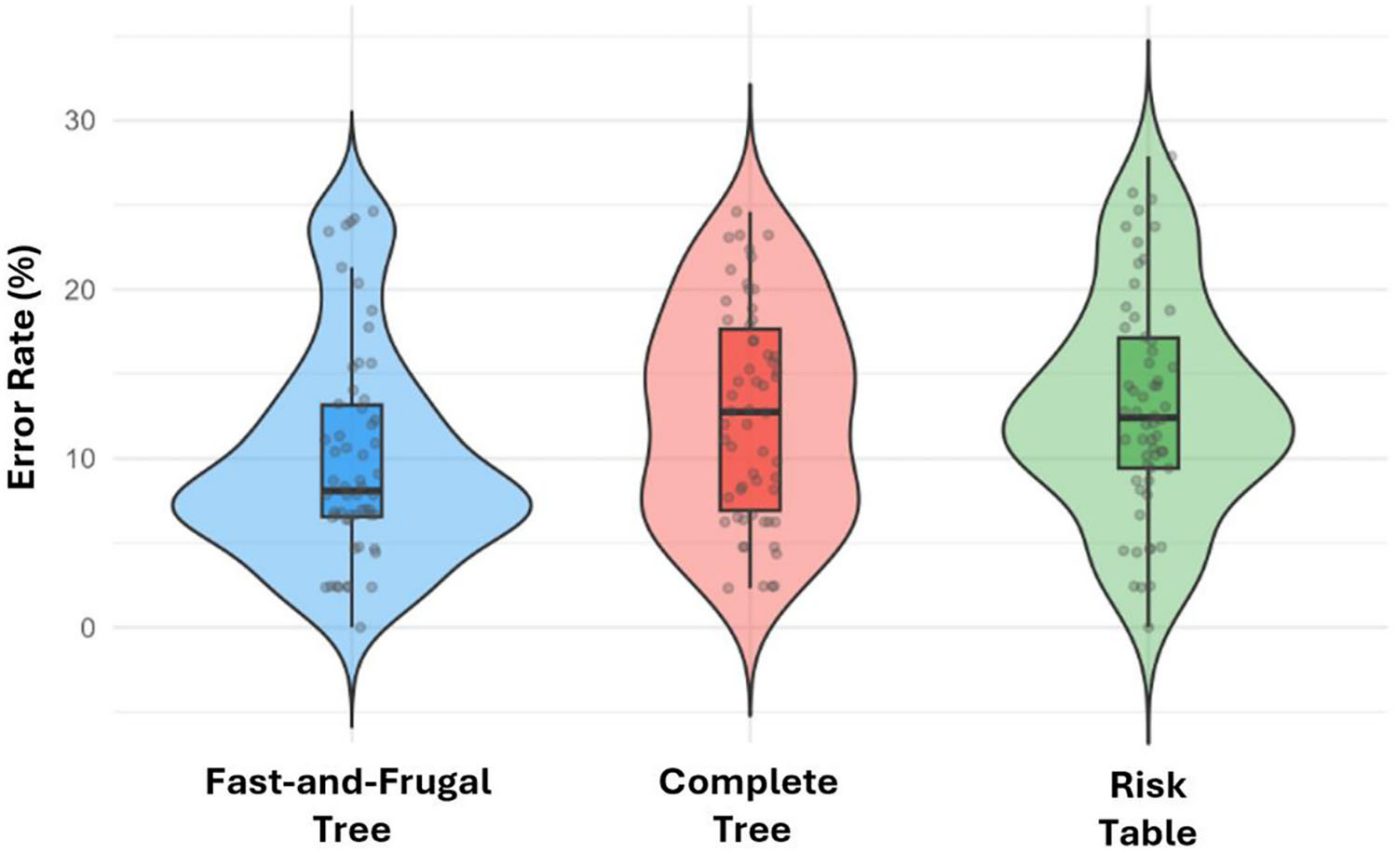
Application error rate (per participant, across trials) for the three conditions.

Figure 8 shows the application error rate (per trial). Error rate was lower for the fast‐and‐frugal tree (9%, SD = 5%) than for the complete tree (14%, SD = 7.9%) and the risk table (15%, SD = 7.9%). Table 8 provides additional descriptive statistics across participants, which also show lower error rates for the fast‐and‐frugal tree.

**TABLE 8:** Application error rates (in %), across participants.

	*N*	Mean (%)	Median (%)	SD (%)	Min (%)	Max (%)
Fast‐and‐frugal tree	58	10.1	8.1	6.3	0.0	24.6
Complete tree	58	12.6	12.7	6.3	2.3	24.6
Risk table	58	13.1	12.4	6.6	0.0	27.9

As for feedback viewing time, we again ran a linear mixed‐effects model with crossed random effects for participants and stimuli (9146 observations, 174 participants, 8 stimuli), followed by post hoc analyses of cumulative error rates using Kruskal–Wallis and Mann–Whitney *U* tests. Table 9 provides the mixed‐effects results, which show that error rate was significantly lower for the fast‐and‐frugal tree than for the complete tree and the risk table.

**TABLE 9:** Linear mixed‐effects analysis for error rate.

	*b*	SE	*z*	*p*	OR	95% CI
Fast‐and‐frugal tree (intercept)	−2.449	0.255	−9.61	< 0.001	0.086	[0.052, 0.142]
Complete tree	+0.233	0.112	2.08	0.038	1.262	[1.013, 1.572]
Risk table	+0.288	0.112	2.58	0.010	1.334	[1.072, 1.660]

A likelihood‐ratio test showed the effect of classification model to be statistically significant (*p* = 0.027). Consistently, a post hoc Kruskal–Wallis test found a significant effect of classification model on error rate [*H*(2) = 8.381, *p* = 0.015, *η* ^2^ = 0.037]. Table 10 shows the results of the Mann–Whitney *U* tests, confirming that the fast‐and‐frugal tree was applied significantly more accurately than the complete tree (small effect) and the risk table (small‐medium effect). Again, there was no significant difference between the complete tree and the risk table conditions.

**TABLE 10:** Post hoc Mann–Whitney *U* tests for error rate.

	*W*	*p*	*r*	Effect size
Fast‐and‐Frugal Tree vs. Complete Tree	1299.5	0.035	0.196	Small
Fast‐and‐Frugal Tree vs. Risk Table	1169.0	0.005	0.263	Small‐medium
Complete Tree vs. Risk Table	1624.5	0.753	0.029	No effect

As for the previous dependent variables, there were no significant interactions between the results for error rate and Berlin Numeracy Test score.

Prediction 3 *Fast‐and‐frugal‐tree users have higher memory test scores*.

Table 11 shows that fast‐and‐frugal‐tree users achieved a mean memory test score of 98.0%, with 93.5% of participants achieving a perfect score. Complete‐tree users scored 91.0% on average (79.0% perfect) and risk‐table users scored 82.3% on average (48.4% perfect). The table includes further descriptive statistics.

**TABLE 11:** Memory test scores across participants.

	*N*	Mean (%)	Median (%)	SD (%)	Min (%)	Max (%)	Perfect scorers
Fast‐and‐frugal tree	62	98.0	100.0	8.2	50.0	100.0	93.5%
Complete tree	62	91.0	100.0	23.7	0.0	100.0	79.0%
Risk table	62	82.3	91.5	29.4	0.0	100.0	48.4%

**FIGURE 9:**
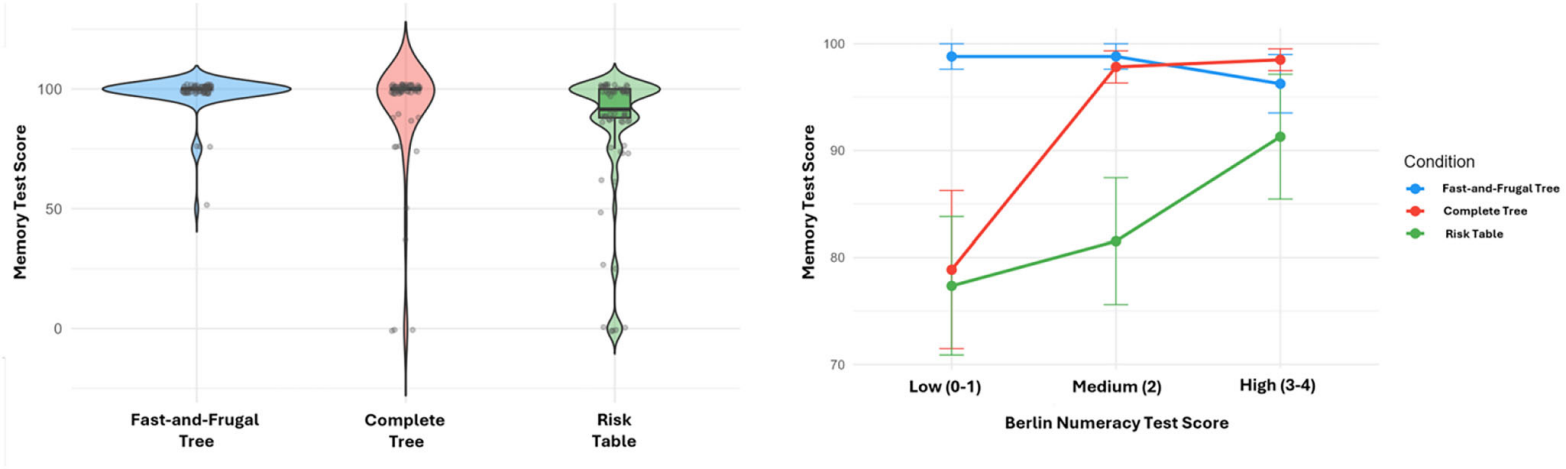
(a) Mean (across participants) memory test scores, with 95% confidence intervals. (b) Interaction between memory test score and Berlin Numeracy Test score can be seen for the complete tree and risk table conditions (red dashed‐and‐dotted line and green‐dashed line respectively); no interaction is seen for the fast‐and‐frugal tree (yellow continuous line).

The mean memory test scores are shown in Figure 9a. Interestingly, Figure 9b shows that memory test scores may interact with scores on the Berlin Numeracy Test, with such an interaction visible for the complete risk and risk table, but not for the fast‐and‐frugal tree.

A post hoc Kruskal–Wallis test revealed a highly significant effect of model on memory test score [*H*(2) = 30.531, *p* < 0.001, *η* ^2^ = 0.156]. In addition, fast‐and‐frugal‐tree users significantly outperformed complete‐tree (*p* = 0.020, *r* = 0.209, small effect) and risk‐table users (*p* < 0.001, *r* = 0.476, medium‐to‐large effect); and complete‐tree users significantly outperformed risk‐table users (*p* = 0.002, *r* = 0.285, small effect). Finally, a post hoc *F*‐test found the interaction between memory test score and Berlin Test Numeracy score to be significant [*F*(2,180) = 3.166, *p* = 0.045].

## 5 Discussion: Contributions, Limitations, and Challenges

### 5.1 Contributions

Breiman et al. 1984, 2001) intuited that visual models such as trees are transparent to practitioners, and this assumption has stayed largely uncontested (Bertsimas and Dunn 2017; Rudin et al. 2022). An important role of the decision sciences is to empirically test assumptions about human behavior (Kunc et al. 2016; Katsikopoulos 2023) but, as has been pointed out (Pande et al. 2021) and argued here, not enough evidence has yet been produced for Breiman's assumption to uncritically endorse visual models for decision and risk analysis support. Our experiment provided evidence for the transparency of visual models such as trees and tables. Perhaps more importantly, the experiment found that it was the combination of visual presentation with model sparsity, as in the case of fast‐and‐frugal trees (Martignon et al. 2008), that created a jump in transparency. A possible reason for this finding is that sparse models can be contemplated at once and show how variables combine (Lipton 2018; Rudin 2019; Herzog and Franklin 2024).

The effect sizes in our experiment potentially translate into big practical benefits: for example, increases in feedback viewing time over a fast‐and‐frugal tree, were estimated at 21% and 46% for a complete tree and a risk table (Table 6). These numbers extrapolated to a real training context, for example 5000 soldiers stationed in Afghanistan by German armed forces with an average of 55 trials per person, may lead to savings of several hundred hours when switching to a fast‐and‐frugal tree. Similar back‐of‐the‐envelope calculations for application and recall accuracy would be equally encouraging. And our exploratory analysis of the robustness of fast‐and‐frugal‐tree recall to differences in statistical numeracy and risk literacy, as predicted by Huysmans et al. (2011), unique among the three models tested (Figure 9b), suggests further gains for workers in high‐risk professions with below‐average to average numeracy. Finally, an anonymous reviewer similarly and reasonably speculated that fast‐and‐frugal trees might be robust to absence of (outcome or process) feedback, a condition that might obtain on the job.

An intriguing, anecdotal finding may also be worthy of future investigation: note that all three models can be viewed as implementing the simple classification rule “If there is one occupant and at least one more attribute pointing to a hostile vehicle, then shoot; otherwise, let pass.” In the debriefing session, some participants did indeed report having used this reformulation. Interestingly, just one fast‐and‐frugal‐tree user did so compared to eight and nine users for the complete tree and the risk table respectively. This finding could be related to the ideas of Lipton (2018), Rudin (2019), and Herzog and Franklin (2024), that sparse models can be contemplated at once, thus decreasing the necessity to reformulate them. This, and related possibilities, should be investigated more directly with process‐tracing methods (Ericsson and Simon 1993).

### 5.2 Limitations and Challenges

The experiment employed an ecologically valid task, filling an empirical gap on the transparency of visual sparse models (Huysmans et al. 2011). The peacekeeping task of threat classification is a high‐stakes, critical, high‐pressure and stressful, difficult task, for which a visual sparse model is known (Keller and Katsikopoulos 2016).

But while the experiment's external validity is relatively high for a controlled lab setting, there are some trade‐offs between internal quality of evidence and external correspondence to reality. For example, to keep the experiment manageable for the participants, we did not present the combinations of attribute values according to the frequencies with which they would be encountered in the field, as this would have extended the experiment to hundreds, possibly thousands of trials. This should be further investigated in future studies. Studies should also include a more nuanced set of actions beyond “shoot” and “do not shoot”, as soldiers on the job have access to graduated force‐escalation (although see Keller et al. 2014, for an integration of fast‐and‐frugal trees with concrete action sequences). Finally, our priority of securing a large number of participants led to a convenience sample of participants with little or no knowledge of the task ecology.

Beyond improving methods, follow‐up research can also be inspired by theory. A key challenge for future research is to try to understand *why* and *when* visual sparse models such as the fast‐and‐frugal tree are more transparent than other visual models such as the complete tree and the risk table. Is it merely model sparsity that drives differences? Which cognitive processes might be activated by fast‐and‐frugal trees that increase transparency? Studies that open the black box of behavior can help move research to *cognitive* decision and risk analysis (Bhashyam and Montibeller 2016; Pande et al. 2021; Katsikopoulos 2023). Intuitive theories of transparent decision and risk analysis support deserve such studies.

## Conflicts of Interest

The authors declare no conflicts of interest.
